# Right Heart Function in Cardiorenal Syndrome

**DOI:** 10.1007/s11897-022-00574-x

**Published:** 2022-09-27

**Authors:** Tilmann Kramer, Paul Brinkkoetter, Stephan Rosenkranz

**Affiliations:** 1 Klinik III Für Innere Medizin, Herzzentrum Der Universität Zu Köln, Köln, Germany; 2 Cologne Cardiovascular Research Center (CCRC), Klinikum Der Universität Zu Köln, Köln, Germany; 3 Klinik II Für Innere Medizin, Nephrologie, Universität Zu Köln, Köln, Germany; 4 Center for Molecular Medicine Cologne (CMMC), Universität Zu Köln, Köln, Germany

**Keywords:** Cardiorenal syndrome, Right heart function, Right heart failure, Pulmonary hypertension, Pulmonary arterial hypertension, Venous congestion, Renal dysfunction, Biomarkers, Treatment approaches

## Abstract

**Purpose of Review:**

Since CRS is critically dependent on right heart function and involved in interorgan crosstalk, assessment and monitoring of both right heart and kidney function are of utmost importance for clinical outcomes. This systematic review aims to comprehensively report on novel diagnostic and therapeutic paradigms that are gaining importance for the clinical management of the growing heart failure population suffering from CRS.

**Recent Findings:**

Cardiorenal syndrome (CRS) in patients with heart failure is associated with poor outcome. Although systemic venous congestion and elevated central venous pressure have been recognized as main contributors to CRS, they are often neglected in clinical practice. The delicate hemodynamic balance in CRS is particularly determined by the respective status of the right heart.

**Summary:**

The consideration of hemodynamic and CRS profiles is advantageous in tailoring treatment for better preservation of renal function. Assessment and monitoring of right heart and renal function by known and emerging tools like renal Doppler ultrasonography or new biomarkers may have direct clinical implications.

## Introduction

Cardiorenal syndrome (CRS) is commonly diagnosed in patients with heart failure (HF) and concomitant chronic kidney disease (CKD). Based on a cardiopulmonary-renal cross talk [1•, 2], acute or chronic dysfunction of one organ impairs the function of the respective other organ [3, 4]. While renal dysfunction in HF has traditionally been considered to result from decreased renal perfusion and associated neural and hormonal changes, recent evidence suggests that rather persistent venous congestion represents a major contributor [5, 6, 7•]. Right ventricular (RV) function plays a key role in preventing CRS in HF and pulmonary hypertension (PH) as the heart aims at compensating the respective state by balancing pre- and afterload [1•]. In right heart failure (RHF), elevated central venous pressure (CVP) leading to venous congestion by backward transmission, was identified to initiate a vicious cycle of hormonal and endothelial activation, hepatic dysfunction, ascites, increased intra-abdominal pressure (IAP), intestinal mucosal ischemia, inflammation, oxidative stress, excessive renal tubular sodium reabsorption, and volume overload, leading to further RV stress [1•, 2, 7•, 8]. Renal congestion caused by HF- or PH-mediated RHF results in renal edema, increased interstitial pressure, tubular compression, and intracapsular tamponade, which may further aggravate back pressure and thus decrease renal blood flow (RBF) and glomerular filtration rate (GFR) [1•, 1, 10, 11]. In situation of decreased renal perfusion, the amount of glomerular blood filtered increases (filtration fraction = GFR/RBF) to maintain GFR. Increase in filtration fraction leads to proximal nephron sodium retention. Additionally, proximal nephron sodium retention leads to a lower fraction of sodium and chloride in the tubular ultrafiltrate at the level of macula densa, leading to neurohormonal activation and worsening renal function (WRF) [4, 12]. The pathophysiology of CRS as a consequence of right heart dysfunction remain poorly understood, which creates an urgent need for biomarkers, diagnostic tools, and interventions to improve renal outcomes [13].

## Study Selection

The aim of this systematic review article was to review the available literature on the meaning of right heart function in CRS “type 1” and “type 2” with a special focus on implications for current diagnostic and therapeutic management. Additionally, we provide an update on clinical and pathophysiological findings regarding CRS and right heart function. Therefore, we performed a comprehensive computerized literature search through multiple Medline searches on the PubMed database using MeSH terms and keywords. Searched terms included a combination of either “cardio- renal syndrome” and/or “pulmonary hypertension” and/or “pulmonary arterial hypertension” and/or “heart failure” an/or “decompensated heart failure” plus each of the following: “biomarker,” “central venous pressure,” “chronic kidney disease,” “classification,” “definition,” “diagnosis,” “heart failure,” “hemodynamics,” “interactions,” “kidney dysfunction,” “kidney failure,” “kidney injury,” “left heart failure,” “management,” “mortality,” “nephropathy,” “outcome,” “pathophysiology,” “prognosis,” “renal dysfunction,” “renal failure,” “renal function,” “renal insufficiency,” “right heart failure,” “right heart function,” “right-sided heart failure,” “survival,” “therapy,” “treatment,” “venous congestion” for articles published before January 2022. In addition to this search, reference lists of review articles were manually searched to identify other possible eligible references. Studies considered for our review included randomized controlled trials, prospective studies, retrospective studies, review articles, and case reports. Studies with unavailable full text or inaccurate data extraction were excluded.

## Classification of Cardiorenal Syndrome

While cardiac dysfunction impairs renal function, renal dysfunction can also lead to numerous harmful effects on the heart [14], and improvement in renal function can lead to cardiac reverse remodeling [15]. Thus, direct and indirect effects of the respective diseased organ cause a combined disorder of both organ systems by a complex combination of neurohormonal feedback mechanisms [16]. For this reason, the current definition by the “Acute Dialysis Quality Initiative” phenotyped CRS into 2 major groups: CRS and reno-cardiac syndrome, depending on the respective primary origin of the disease process. This classification distinguishes 5 subtypes according to disease severity and sequential organ involvement to enable a more precise and logical approach [3, 16, 17•]. The CRS definition includes acute or chronic conditions, whereby the primary failing organ can be either the heart or the kidney [3, 14, 16]. The exception is subtype 5, which represents a systemic condition affecting both organs simultaneously (Table 1).

**Table 1:** Classification of cardiorenal syndrome based on the “Consensus Conference of the Acute Dialysis Quality Initiative” [3, 16, 17•]

CRS phenotype	Nomenclature	Description	Clinical examples
Type 1	Acute	HF resulting in AKI	ACS resulting in cardiogenic shock and AKI, AHF resulting in AKI
Type 2	Chronic	Chronic HF resulting in CKD	Chronic HF
Type 3	Acute renocardiac syndrome	AKI resulting in AHF	HF in the setting of AKI from volume overload, inflammatory surge, and metabolic disturbances in uremia
Type 4	Chronic renocardiac syndrome	CKD resulting in chronic HF	LVH and HF from CKD-associated cardiomyopathy
Type 5	Secondary	Systemic process resulting in HF and renal failure	Amyloidosis, sepsis, cirrhosis

ACS, acute coronary syndrome; AHF, acute heart failure; AKI, acute kidney injury; CKD, chronic kidney disease; CRS, cardiorenal syndrome; HF, heart failure; LVH, left ventricular hypertrophy

In contrast, the previous terminology did not allow to identify and fully characterize the chronology of the pathophysiological interactions that characterize a particular type of combined cardiac/renal disease [16].

The current definition was established to facilitate a precise clinical characterization of CRS and to provide a basis for the development of new diagnostic and treatment approaches [17•]. Since the different subtypes of the current definition often overlap and may change dynamically during disease progression, deriving therapeutic recommendations remain clinically challenging. Functional assessments of cardiac and renal function for exact assignment to the different subtypes are still scarce [4, 17•].

## Pathophysiological Evolution from Low Perfusion to Renal Congestion

It has been traditionally postulated that reduced cardiac output (CO) leads to diminished renal perfusion and neurohormonal activation representing the main drivers for the development of CRS in HF with reduced ejection fraction (HFrEF) [4, 5, 6, 7•, 18, 19]. The right heart function is primarily important to maintain sufficient RV filling by adequate preload [4]. It has been assumed that a cardiac index (CI) of at least 1.5 l/m^2^min is necessary to maintain sufficient forward transmission for adequate renal perfusion [4, 19]. The impairment of renal arterial blood flow by either low CO and/or increased peripheral vascular resistance is considered to activate the renin–angiotensin–aldosterone system (RAAS), sympathetic nervous system, and release of arginine vasopressin [4, 18]. Activation of these neurohormones causes systemic and renal vasoconstriction and increased sodium and water retention in the kidneys to compensate arterial underfilling and reduced preload, but at the expense of increased systemic vascular resistance and higher plasma volume, thus leading to worsening HF [13, 18].

Recently, venous congestion has been recognized as important contributor to renal dysfunction in CRS [7•]. Regardless of whether left or right HF is present, venous congestion, increased CVP, and increased renal venous pressure appear to be the major hemodynamic contributors to CRS [1•, 5, 7•, 20•, 21]. The importance of systemic venous congestion in terms of WRF is supported by the finding that deterioration of kidney function occurs more frequently in HF with preserved ejection fraction (HFpEF) than in HFrEF [4, 22].

These findings place the pathophysiological focus of CRS more on right heart function. RV dysfunction, which is also frequently present in patients with left-sided heart disease, leads to backward transmission of elevated filling pressures, resulting in increased CVP and renal venous congestion. Due to complex RV/LV interactions, RHF results in underfilling of the left ventricle (LV) and systemic low-output and in advanced cases to cardiogenic shock [4]. Thus, right heart dysfunction plays a pivotal role for secondary organ damage. Acute RHF may occur because of rapidly increased RV afterload through pulmonary embolism, decompensated RHF/LHF, or hypoxia [4]. Another cause of acute RHF represents impaired RV contractility in consequence of ischemia, myocarditis, or postcardiotomy shock [4]. Chronic RHF is mainly due to chronically elevated pulmonary artery pressure (PAP) and/or pulmonary vascular resistance (PVR), i.e., increased RV afterload in PH. The downstream disturbances in cardiorenal interactions are likely to be similar regardless of whether the primary cause was right-sided or left-sided HF [4].

PH is a hemodynamic condition defined by an elevated mean PAP to ≥ 25 mmHg at rest, measured invasively by right heart catheterization (RHC) [23]. A revised definition was proposed at the 6th World Symposium on Pulmonary Hypertension (WSPH) in 2018, suggesting a redefined threshold of > 20 mmHg at rest [24].

Any kind of PH can contribute to RHF [13, 25] and thus to the development of CRS [4, 13, 26]. PH due to left heart disease represents the most common cause of RHF, and the pathophysiology of CRS in this group is comparable to that in left heart failure (LHF) [13]. Data on the prevalence of CRS in patients with PH and isolated RHF remain scarce [13]. However, PH is associated with impaired kidney function leading to poorer outcome [1•, 20•, 26–30]. Similar to LHF, isolated RHF in PH often results in renal venous congestion and arterial hypoperfusion with subsequent loss of GFR [13, 20•]. Conversely, renal dysfunction may also contribute to aggravation of PH, since cardiopulmonary hemodynamics are crucially dependent on the regulation of fluid homeostasis. Elevated CVP leads to release of inflammatory mediators, neurohormones, and activation of endothelial cells, which may further deteriorate heart and kidney function [13, 21, 31]. Circulatory factors excreted in the kidney are involved not only in the pathogenesis of local structural glomerular and interstitial damage but also in pulmonary inflammation following renal injury [1•, 2, 32]. In PH, the right heart function is elementary to cope with elevated PVR by balancing pre- and afterload resulting in water and salt retention and venous congestion [1•]. LV output is potentially also reduced in PH, as reduced RV output and dilatation frequently lead to leftward shift of the interventricular septum and altered LV geometry, resulting in impaired filling and output despite preserved LVEF [1•, 4, 13, 33–35]. Consequently, forward transmission can also be diminished in PH, leading to reduced renal arterial pressure and thus contributing to the development of CRS [4, 6, 33, 36]. In PAH, reduced CI rather than increased RAP was an independent predictor for WRF [1•, 20•], while in a study on RHF due to miscellaneous PH classes with preserved LVEF elevated RAP rather than CI independently predicted GFR [13, 37]. These results indicate that in PAH, reduced CI and subsequent renal hypoperfusion might be the key hemodynamic drivers of GFR loss and may precede deleterious effects on kidney function of augmented RAP and renal venous congestion during worsening right heart function [20•]. Patients with PH due to left heart disease might be more prone to renal venous congestion similar to the LHF phenotype [13]. Differentiation between hemodynamic profiles may be advantageous in tailoring treatment for preservation of GFR in HF and various forms of PH.

**Fig. 1:**
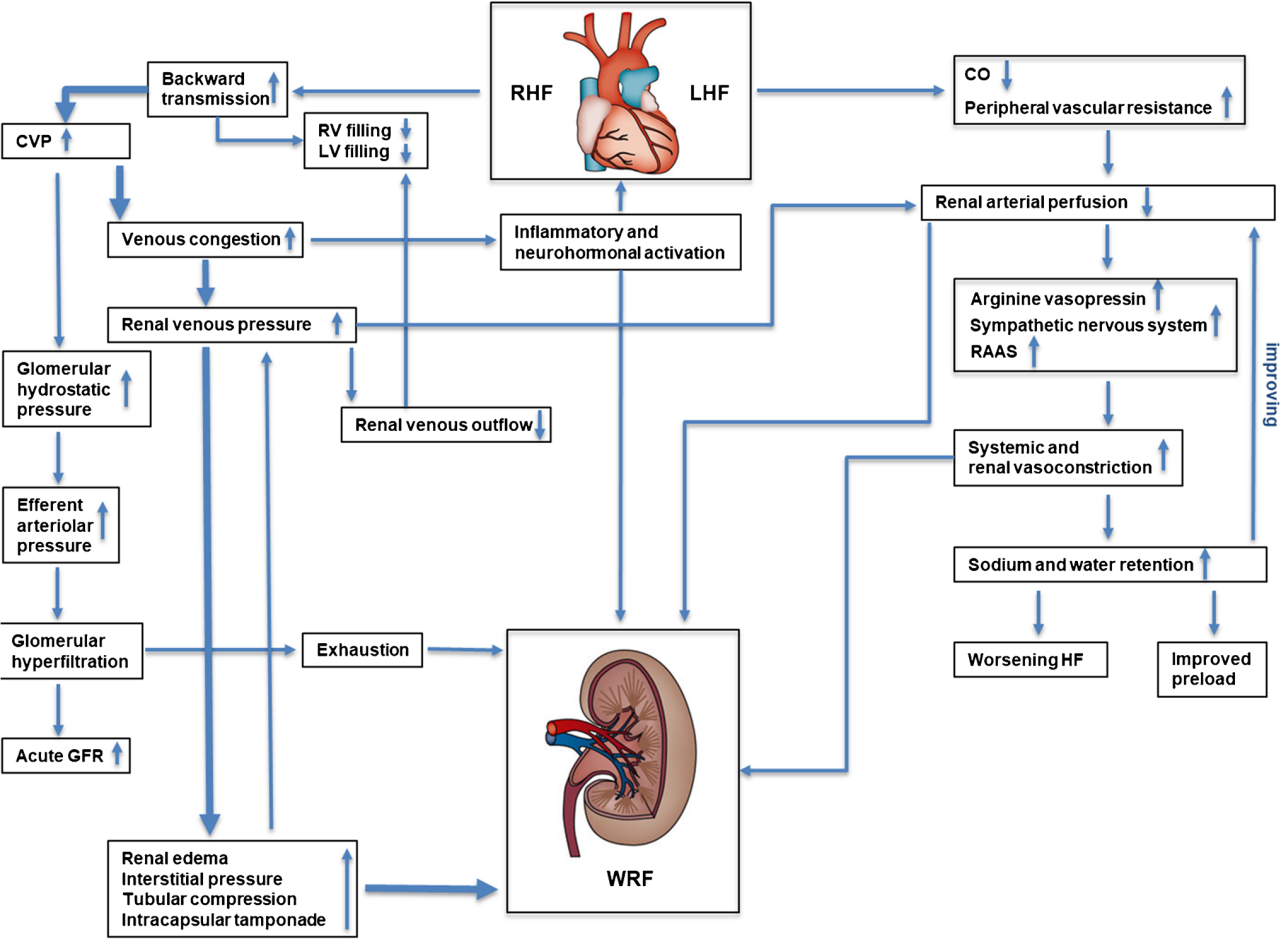
Hemodynamic cardiorenal interactions in left and right heart failure. **Right side:** traditional “low flow” hypothesis of the development of worsening renal function in heart failure due to renal vasoconstriction and hypoperfusion leading to tubular hypoxia and necrosis. **Left side:** concept of “congestive nephropathy” primarily leading worsening renal function in heart failure according to current expert opinion. Heart failure-induced backward transmission results in increased central venous pressure and renal venous congestion with subsequent impaired renal function. CO, cardiac output; CVP, central venous pressure; GFR, glomerular filtration rate; HF, heart failure; LHF, left heart failure; LV, left ventricle; RAAS, renin–angiotensin–aldosterone system; RHF, right heart failure; RV, right ventricle; WRF, worsening renal function. Adapted from Schefold, J. C. et al. (2016) Heart failure and kidney dysfunction: epidemiology, mechanisms and management. Nat. Rev. Nephrol. 10.1038/nrneph.2016.113 and from Rosenkranz S, Howard LS, Gomberg-Maitland M, Hoeper MM. Systemic Consequences of Pulmonary Hypertension and Right-Sided Heart Failure. Circulation 2020;141:678–693

Elevated CVP initially leads to a slightly increased GFR through increased glomerular hydrostatic pressure due to elevated proximal peritubular capillary pressure, resulting in increased efferent arteriolar pressure [6, 7•, 13, 38, 39]. After glomerular hyperfiltration is exhausted, GFR progressively worsens [7•] due to renal edema, increasing interstitial pressure, tubular compression, and intracapsular tamponade, which may further aggravate back pressure and thus decrease renal perfusion pressure and GFR [1•]. The term “congestive nephropathy” was suggested for a potentially reversible subtype of renal dysfunction caused by reduced renal venous outflow and increased renal interstitial pressure [7•]. Based on increased CVP, another contributor to WRF is ascites and increased IAP [9]. Immediate reduction of IAP by large volume removal improves GFR in decompensated HF [40], which is why ascites and IAP assessment could be integrated into the diagnostic and therapeutic workflow. Pathophysiological interactions are depicted in Fig. 1.

## Diagnosis of Right Heart Dysfunction and Cardiorenal Syndrome

### Hemodynamics

RHF can occur in progressive or acutely decompensated PH, leading to chronic or acute kidney damage and thus contributing to CRS. In combined RHF with concomitant acute renal failure, deterioration of either organ function may converge in a vicious circle leading to refractory congestive RHF [1•]. In CRS prevention, precise measurements of right heart function are gaining importance. The most accurate way to determine right heart function is RHC [23, 24, 41]. A cutoff value of 15 mmHg for pulmonary arterial Wedge pressure (PAWP) distinguishes between precapillary (≤ 15 mmHg) and postcapillary (> 15 mmHg) PH. This distinction is important since postcapillary PH often occurs in LHF and primarily requires optimized therapy of the left heart condition, while in precapillary PH such as PAH or CTEPH, special treatments are available to improve RHF [23]. Precise hemodynamic assessment is of utmost importance for correct diagnosis und therapeutic management potentially affecting CRS. In experienced hands, RHC is safe and could be warranted in selected difficult-to-treat CRS patients to detect and treat subclinical congestion while avoiding intravascular underfilling [42].

Transthoracic echocardiography represents a more convenient and noninvasive assessment tool of right heart function, which can be performed in nearly every clinical situation. Specific echocardiographic measurements provide information about right heart function in CRS and helps to identify those patients who might require RHC [23]. Echocardiographic signs of right heart strain increase the risk of RHF and systemic venous congestion contributing to CRS. Therefore, control of congestion has been proposed as a central goal in HF treatment [5, 6, 43•]. Assessment of the diameter of the inferior vena cava and its respiratory variability is of particular importance as it correlates positively with CVP and RAP [44, 45].

Renal Doppler ultrasonography represents a relatively new approach to evaluate renal congestion and to guide therapy in patients with left- or right-sided HF [43•, 46•]. Based on Doppler renal venous flow, identification of altered intrarenal venous flow (IRVF) patterns is used to predict adverse outcome and to monitor diuretic response in HF [43•, 47, 48•, 49]. IRVF patterns depend on RAP and the mean circulatory filling pressures [4, 47, 48•]. Backward transmission in RHF results in elevated RAP, which is transmitted into the renal vein with consequently increased pulsatility and change in IRVF patterns, reflecting the renal vasculature’s response to elevated intrarenal pressure within the rigid renal capsule [4, 50]. Compared to renal resistance index, the IRVF pattern demonstrates higher prognostic impact [47], confirming renal venous congestion rather than hypoperfusion as the predominant component in CRS development [1•, 4]. Renal venous congestion index (RVSI) was described as a dimensionless continuous ratio that reflects the complete continuum of renal venous congestion by indicating the fraction of the cardiac cycle during which there is no renal venous outflow [43•, 46• ]. In PH, RVSI correlates with right heart and renal function [46•]. Its measurement represents a simple and noninvasive approach for the assessment of renal congestion [43•, 46•] and provides additional prognostic information to stratify the risk of RHF in PH [46•], which in turn can result in CRS [17•].

### Biomarkers

Various biomarkers reflecting neurohormonal disorders, myocardial stress/injury, inflammation, oxidative stress, and renal clearance/injury have been considered for their diagnostic and prognostic value in HF and CRS [13, 17•, 51]. Since progressive deterioration in right heart function is associated with slow progressive degenerative processes potentially resulting in CKD, sensitive biomarkers are needed [1•, 52].

### Cardiac Biomarkers

#### Brain Natriuretic Peptide and N-Terminal Pro-brain Natriuretic Peptide

Measurement of brain natriuretic peptide (BNP) or N-terminal pro-BNP (NT-proBNP), which is directly associated with congestion, is supported by current HF and PH guidelines [23, 53] and plays a central role for diagnosis and prognosis of CRS in HF [17•]. BNP/NT-proBNP serum levels in HF with CRS are higher compared to patients with preserved kidney function [4, 54, 55•], which may be explained by several mechanisms including impaired renal excretion and volume overload [4, 56–58]. In patients with GFR < 60 ml/min/1.73m2, BNP/NT-proBNP serum concentrations should be interpreted with caution and is only of limited use in diagnosing HF and monitoring treatment response especially when HF and volume overload are present [59]. Nevertheless, elevation of BNP/NT-proBNP in HF with already existing renal dysfunction is also associated with poorer prognosis [54, 55•]. Currently, BNP/NT-proBNP measurement is not recommended for prevention or treatment of acute kidney injury (AKI) [45].

#### Cardiac Troponin T and Galectin-3

Cardiac troponin T and galectin-3 represent useful cardiac biomarkers which are elevated in CRS [4, 58, 60, 61] and prognostically relevant in HF [4, 60, 61]. However, elevated troponin T serum levels are unspecific for renal damage and occur in most patients with advanced renal dysfunction [54, 55•] and thus must be interpreted with caution when diagnosing acute coronary syndrome in these patients [59]. Determination of troponin T is only recommended on admission to exclude acute myocardial injury; albeit, it is elevated in most acute HF patients [53].

Expression of galectin-3 is linked to fibrosis [55•, 62, 63], including renal [51, 64, 65] and cardiac remodeling and progression of HF [55•, 63, 66–69]. Thus, galectin-3 potentially provides information about the pathophysiology of the underlying renal dysfunction and its progression in HF [55•, 70–72]. High galectin-3 plasma levels are associated with increased risk of renal dysfunction [55•, 73, 74]. In HF, elevated galectin-3 is also linked to impaired kidney function and higher mortality [51, 70]. Through its ability in early detection of WRF, it may be used for risk estimation of CRS progression and potentially as therapeutic target [55•, 75].

### Renal Biomarkers

#### Cystatin C

Cystatin C (CysC) is a protease occurring in all nucleated cells [51, 55• ]. In contrast to creatinine, it is filtered freely and then reabsorbed but not secreted in renal tubules [51, 55•]. It was assumed that assessment of CysC could be superior to serum creatinine as it is independently excreted from the respective muscle mass [76]. CysC represents a promising alternative endogenous filtration biomarker for monitoring renal function [51, 59, 76, 77] and a prognostic indicator in HF with normal renal function [51, 55•, 78–80]. However, assessment of CysC provides no information about the different pathomechanisms of CRS [51].

#### Albuminuria

Albuminuria is associated with increased cardiovascular disease (CVD) risk and is prognostically relevant for CKD progression [59]. In HF, albuminuria is associated with poorer outcome [4, 81, 82]. Albuminuria indicates damage to the glomerular filter, primarily due to dysfunction of podocytes, which are crucial for maintaining glomerular filter barrier [83]. In addition, endothelial dysfunction, inflammation, elevated glomerular pressure, or atherosclerosis contribute to increased albumin excretion [55•, 84, 85]. Albuminuria also occurs in anomalous renal microcirculation [55•, 86] and can reflect impaired renal hemodynamics, such as renal venous congestion caused by RHF and increased CVP [6, 55•]. Accordingly, albuminuria provides information about the pathophysiology leading to WRF [55•, 84]. The assessment of albuminuria integrates urinary creatinine levels to calculate the ratio between urinary albumin and creatinine (UACR) [55•]. Microalbuminuria, defined by UACR between 30 and 300 mg/g [85], is common in HF and associated with worse prognosis [55•, 81, 82, 87].

#### Neutrophil Gelatinase–Associated Lipocalin

Neutrophil gelatinase–associated lipocalin (NGAL) is a protein that is freely filtered through the glomerulus and completely reabsorbed in the proximal part of the tubule [55•]. Under normal conditions, its concentration in urine and serum is very low [51, 55•, 88]. In proximal tubular injury, NGAL urinary level rises above the normal range [4, 89], because it cannot be completely reabsorbed due to tubular damage [55•]. High NGAL levels occur in HF with and without renal dysfunction [4, 51, 58, 90] and predict WRF, particularly in acute decompensated HF [51], and adverse clinical outcome [55•, 91, 92], also in CRS patients [4, 93]. However, the diagnostic accuracy of NGAL is not without controversy and may be affected by confounding factors such as sepsis, inflammation, anemia, hypertension, hypoxemia, and cancer [55•, 88].

#### N-Acetyl Beta Glucosaminidase

N-Acetyl beta glucosaminidase (NAG) is a lysosomal enzyme of the proximal tubule cells, which is excreted into urine if tubular damage with subsequent disruption of lysosomal integrity occurs [51, 55•, 89, 94, 95]. NAG serum levels are gradually elevated in HF with preserved or reduced renal function and are associated with poorer prognosis, independently from GFR but dependent on LVEF [51, 55•, 96–98]. Increase in NAG is predictive for AKI in patients with normal and worsening kidney function and for impaired survival in patients with preexisting AKI [51, 95, 99]. Despite limited specificity [51, 96], NAG is a promising prognostic biomarker for CRS and could also represent a potential therapeutic target since it decreases in response to diuretic-induced decongestion similar to BNP/NT-proBNP [51, 100], which is particularly interesting since venous congestion is the major pathophysiological driver of CRS [7•, 7, 51].

#### Kidney Injury Molecule-1

Kidney injury molecule-1 (KIM1) is a glycoprotein which is expressed in proximal tubule cells and excreted in the urine after tubular injury [55•, 95]. High urinary KIM1 concentrations predict poorer prognosis including WRF in HF independent of GFR [55•, 96–99] but dependent on LVEF in terms of quantitative characteristics [51, 98]. KIM1 levels correlate with BNP/NT-proBNP and are sensitive to volume fluctuations, reflecting their dependence on congestive clinical states and making it an excellent target for diuretic management of CRS and a possible biomarker for CRS phenotyping [51, 98, 100].

#### Dickkopf-3

Dickkopf-3 (DKK3) is a stress-induced, tubular epithelial-derived profibrotic glycoprotein which predicts WRF [101–103]. Increased urinary DKK3 levels identify patients at high risk for short-term GFR decline regardless of the respective cause of kidney injury and are associated with tubulointerstitial fibrosis [102–104]. Although data on the meaning of urinary DKK3 levels in HF are currently pending, DKK3 might represent a promising future biomarker in CRS. Potentially, elevated DKK3 urinary levels are indicative for active renal fibrosis, contributing to poorer outcome in CRS. Hence, urinary DKK3 might serve as a biomarker to monitor CKD progression which may be useful for clinicians to monitor treatment effects and guide therapeutic adjustments [102–104].

Together, there are numerous promising novel biomarkers to monitor kidney function in CRS. However, none of these is specific to impaired right heart function or CRS, making it difficult to identify impaired right heart function as the main contributor to CRS [4]. Therefore, CRS phenotyping must integrate information from biomarkers, hemodynamics, and imaging modalities and should always be interpreted in the clinical context.

### Therapeutic Approaches

#### Diuretics, Volume Optimization, and Ultrafiltration

Since elevated CVP and venous congestion were recognized as main drivers for CRS, diuretics represent the initial drug of choice in decompensated HF associated with inadequate fluid retention to decrease volume overload and to improve cardiorenal hemodynamics [42, 53, 105–107]. Reduction of RV overload, CVP, and renal venous pressure lead to increased renal perfusion and to improved RV/LV interaction, cardiac and kidney function [4, 107]. Loop diuretics are frequently used for fast natriuresis with subsequent extracellular volume reduction [107–109]. There are no differences in outcome, symptom relief, or changes in renal function when loop diuretics are administered as bolus or continuous therapy [4, 42, 106]. High-dose compared to low-dose administrations resulted in a faster relief of congestion with a transient reduction in GFR [42, 106]. The addition of non-loop diuretics might be reasonable to maintain natriuresis without compromising GFR [4, 110, 111]. The level of care for HF patients with CKD should be the same as for those without CKD, but any escalation of therapy and/or clinical deterioration should prompt monitoring of GFR and serum potassium concentration [61]. Acetazolamide as a potent inhibitor of proximal tubular sodium reabsorption could represent another interesting option in decongestive treatment strategies, as targeting sodium reabsorption in the proximal tubules implies potential benefits in HF. The ADVOR trail is currently investigating whether acetazolamide in combination with loop diuretic therapy can improve outcome and decongestion in acute HF with fluid overload [112, 113]. Acetazolamide may be considered if loop diuretic response remains insufficient [53].

During diuretic treatment in acute congestive HF with WRF, intensive volume depletion initially results in increases in serum creatinine and biomarkers of tubular injury (NAG, KIM1, NGAL), while renal function improves over time, suggesting that benefits of decongestion may outweigh transient increases in serum creatinine or tubular injury markers at treatment start [1•, 114]. Caution is advised in decompensated HF without congestion and excessive diuresis, both of which may be associated with reduced RV preload and hence impairment of CO, thus resulting in intravascular hypovolemia, hypotension, and decreased diuresis and natriuresis [4]. When the RV is collapsed, careful volume loading can be beneficial until RV is adequately filled. Further volume expansion can induce adverse effects and should be avoided, especially in patients with mean arterial pressure less than 60 mmHg [13]. Nephrotoxic agents should be temporary avoided [59], regardless of whether decompensated HF is congestive or non-congestive [13]. Since ascites contributes to WRF through increased IAP, paracentesis potentially represents a viable treatment option for acute hemodynamic improvement. Ultrafiltration is another decongestive strategy in decompensated HF and concomitant renal dysfunction, although it does not lead to better outcome or renal function compared to pharmaceutical care only [42, 111]. Ultrafiltration is associated with a higher rate of adverse events [111] and is not an effective therapy in CRS [108]. Future studies on individually titrated ultrafiltration patients are warranted [45, 115], especially in HF and CRS.

#### Diuretic Resistance

Since decongestion is the key treatment strategy to reduce venous congestion in decompensated HF-associated CRS [4, 42, 116–118], special attention should be paid to the potential development of diuretic resistance, which is predictive for poor outcome in CRS and HF [4, 51, 119–121]. Diuretic resistance is defined as reduced diuretic response resulting in inadequate relief of edema, thereby missing the therapeutic target [4, 122]. Decongestion strategies in CRS patients with diuretic resistance remain clinically challenging, as this is often associated with significant reduction of GFR and CO, which results in diminished delivery of diuretics to the tubules [4, 51, 107, 119]. Another potential contributor to impaired delivery of protein-bound loop diuretics is hypoalbuminemia which is frequent in advanced HF [4, 107]. Further causes are insufficient dosage of diuretics or inadequate substrates (sodium and chloride) at the renal tubules [4, 107, 119]. In addition, activation of the RAAS by diuretic-inherent effects results in reduced renal arterial flow and tubular secretion [51, 123]. Further contributors to diuretic resistance are nephron remodeling due to prolonged use of loop diuretics [4, 124] and diuretic braking phenomena by hemodynamic and neurohormonal mechanisms including RAAS activation and afferent arteriolar vasoconstriction [4, 51, 125, 126], which lead to diminished natriuresis to preserve intravascular volume in response to increased, diuretics-induced sodium excretion [4, 51, 107, 127]. In CRS, renal dysfunction leads to impaired release of diuretics into the tubular lumen, and sodium excretion is diminished because of reduced filtration [4, 128, 129]. Diuretic resistance is indicative of HF-induced renal dysfunction and less dependent on GFR, suggesting that determination of diuretic resistance is helpful to identify CRS patients [51]. The diuretic dose–response curve in HF patients typically has a sigmoid shape and demonstrates a rightward and downward shift [130]. Given the dependency on RBF, higher doses of loop diuretics might be necessary in CRS. The soon to be published ADVOR trial with acetazolamide as a combinatorial diuretic treatment will clarify whether this strategy is superior to conventional loop diuretic treatment in acute HF [112, 113]. The forthcoming CLOROTIC trial evaluates whether the addition of hydrochlorothiazide to a loop diuretic represents an effective strategy for decongestion in HF [131], since this is recommended for nephrotic patients with diuretic resistance [115]. In two recent trials, the sodium-glucose cotransporter 2 (SGLT2) empagliflozin increased diuresis and demonstrated beneficial effects in acute decompensated HF patients without impairing kidney function [132, 133].

### Renin–Angiotensin–Aldosterone System Inhibitors

RAAS activation is crucially involved in pathophysiological changes contributing to WRF. In HFrEF, reno-protective RAAS inhibitors belong to the standard of care and have demonstrated beneficial effects on cardiovascular outcome [53, 59]. Because CRS and hyperkalemia are common in HFrEF [7•, 134–136], particular attention should be paid to RAAS inhibitors and novel treatment agents [53] for possible interactions with renal function. Indeed, the occurrence of renal dysfunction and hyperkalemia are challenges for RAAS inhibitor therapy in clinical practice [59, 135, 136•]. In HFrEF therapy, RAAS inhibition is linked to WRF, which is associated with poorer outcomes compared to no WRF [137]. However, the use of RAAS inhibitors leads to a reduction of all-cause mortality, which is significantly more pronounced in the presence of WRF than in the group without WRF [137]. This striking difference could be explained by the fact that in a state of reduced kidney function there is more intensive RAAS stimulation [137–139], which in turn causes the outstanding potential for improvement with sufficient RAAS blockade [137].

### Permissive Acute Kidney Injury

In HFrEF, RAAS inhibitors and SGLT2 inhibitors can reduce renal perfusion, possibly followed by an acute decrease in GFR [53]. However, this should not lead to immediate discontinuation of these beneficial treatments [53, 137, 140]. Acute declines in GFR should not be misinterpreted as AKI but rather be understood as “permissive AKI” [140], since the use of the respective agents contributes to better preservation of kidney function longer term and reduced all-cause mortality [137]. The optimal therapeutic strategy includes assessment of the clinical setting in which GFR loss occurs [140]. In the absence of alternative treatable causes (e.g., infections, nephrotoxic co-medication, hypotension), a decline in GFR of 30–40%, e.g., under RAAS inhibitor therapy, should be tolerated and not lead to discontinuation of this outcome-modifying therapy [137, 140].

### Soluble Guanylate Cyclase Stimulators

Vericiguat, a soluble guanylate cyclase stimulator, represents a new treatment option in HFrEF [53] that improved outcome, irrespective of baseline GFR or WRF [136•, 141–143]. Vericiguat may be considered in addition to standard therapy in HFrEF to reduce risk of cardiovascular mortality and hospitalizations for HF [53]. Vericiguat demonstrated no negative effects on renal function, and, thus, there is no need to down-titrate or interrupt therapy if WRF and/or hyperkalemia occur [136•]. Vericiguat appears to be a potential candidate for the prevention of CRS in HFrEF patients, although the exact mechanisms of action on renal function remain speculative and require further research. On the one hand, vericiguat could improve RBFby its positive effects on cardiac and endothelial function, on the other hand, mild blood pressure lowering effects may potentially impair renal perfusion [136•].

### Sodium-Glucose Cotransporter 2 Inhibitors

SGLT2 inhibitors are recommended for the treatment of HFrEF patients with and without type 2 diabetes mellitus [53]. Regardless of the presence of diabetes, SGLT2 inhibitors showed beneficial effects on cardiorenal outcome in HFrEF and HFpEF [108, 144–147]. These cardio-reno-protective benefits render SGLT2 inhibitors a promising drug in CRS prevention and treatment [148]. Selective blockade of the SGLT2 transporter increases renal excretion of glucose and sodium by inhibiting their reabsorption in the renal proximal tubules [149–151]. Reno-protective effects are mainly mediated by reduction in albuminuria, inflammation, hypoxic stress, renal artery stiffness, and restoration of tubuloglomerular feedback [108, 148]. Reinforced diuresis may further contribute to advantageous effects of SGLT2 inhibitors on kidney function [108, 152]. Overall, the safety profile of SGLT2 inhibitors is compelling, with mild genital mycotic infections being the most common but rare adverse event [153]. Euglycemic diabetic ketoacidosis is a rare but serious side effect of SGLT2 inhibitor treatment, occurring mainly in diabetes with insulin deprivation, after surgery [153] or under infectious conditions [154].

### Vasopressin V2 Receptor Antagonists

In HF, RAAS activation leads to elevated vasopressin levels with subsequent impairment of cardiac function, peripheral vasoconstriction, and increased afterload [108]. There is a vasopressin V2 receptor-mediated water retention and, thus, an increase in preload [108, 155, 156]. Tolvaptan is a highly selective vasopressin V2 receptor antagonist with a convincing safety profile that has demonstrated beneficial effects in HF contributing to reduced volume overload, improved symptoms, increased urinary output, and corrected sodium levels without affecting renal function and serum electrolytes through its action on neurohormonal signaling in CRS [108, 155, 157]. Although tolvaptan failed to improve outcome in HF [53, 108, 158], data suggest that it may serve as a potential drug for decongestion in CRS [108, 159], thus promoting renal function by maintaining renal perfusion and avoiding intravascular volume depletion [108, 160]. Tolvaptan can be considered to increase serum sodium and urinary output in patients with persistent hyponatremia and congestion [53].

### Treatment of Pulmonary Hypertension

In PH, the use of diuretics is recommended in patients with fluid retention associated with RHF [23], whereas the initiation of targeted therapies requires precise hemodynamic diagnosis and classification [23]. Targeted treatment results in improved cardiopulmonary hemodynamics and RV function without significant changes in GFR [20•], although most medications were shown to have nephroprotective potential in preclinical or clinical settings [26]. Treatment of PAH with the phosphodiesterase type 5 inhibitor (PDE5i) sildenafil was associated with improved kidney function [1•, 161]. Changes in kidney function could be due to several influencing factors, such as progressive PAH, the more intensive use of diuretics or polypharmaceutical effects. Since there are no dedicated clinical trials that have investigated the impact of PAH-targeted therapy on kidney function, further research is warranted [26]. Despite limited data, treatment with pulmonary vasodilators (e.g., inhaled nitric oxide, prostacyclin, and iloprost) and inotropes resulted in beneficial effects on CO and venous congestion [4, 108, 162].

In LHF, backward transmission of elevated left-sided filling pressure into the lung leads to postcapillary PH [163–166]. The primary strategy to improve cardiopulmonary hemodynamics is optimization of volume status and filling pressures. Although PH is common in HFrEF and HFpEF [167] and is associated with unfavorable cardiorenal outcomes, there is currently no recommendation for targeted PH therapy in both of these entities [23, 168, 169]. Preliminary data suggests that treatment with PDE5i might have advantageous effects in HFpEF and combined post- and precapillary PH [43, 163, 170, 171]. Further studies are needed since there are currently no data on the impact on CRS.

## Conclusions

Systemic venous congestion and elevated CVP in the context of RV dysfunction are major contributors in CRS. Although the crucial contribution of congestion to CRS and its impact on outcomes have been well documented, they often remain neglected in clinical practice. Thus, accurate assessment and the recognition of right heart function in CRS gain in importance. CRS in RHF should be suspected when WRF occurs either in cases of AKI without preexisting renal damage or in acute-on-chronic settings. The delicate hemodynamic balance is crucially affected by the respective status of the right heart. RHF patients are potentially threatened by acute decompensation, which must be prevented with close-meshed and strict volume management aiming at preserving or improving renal function and outcome. Assessment and monitoring of right heart and renal function by known and emerging tools like renal Doppler ultrasonography or new biomarkers may have direct clinical implications. The monitoring and differentiation between hemodynamic and CRS profiles may be advantageous in tailoring treatment for preservation of renal function.
